# Needs, Challenges, and Applications of Artificial Intelligence in Medical Education Curriculum

**DOI:** 10.2196/35587

**Published:** 2022-06-07

**Authors:** Joel Grunhut, Oge Marques, Adam T M Wyatt

**Affiliations:** 1 Schmidt College of Medicine Florida Atlantic University Boca Raton, FL United States; 2 Department of Electrical Engineering and Computer Science College of Engineering and Computer Science Florida Atlantic University Boca Raton, FL United States; 3 Department of Biomedical Sciences Schmidt College of Medicine Florida Atlantic University Boca Raton, FL United States; 4 Department of Population Health and Social Medicine Schmidt College of Medicine Florida Atlantic University Boca Raton, FL United States

**Keywords:** artificial intelligence, AI, medical education, medical student

## Abstract

Artificial intelligence (AI) is on course to become a mainstay in the patient’s room, physician’s office, and the surgical suite. Current advancements in health care technology might put future physicians in an insufficiently equipped position to deal with the advancements and challenges brought about by AI and machine learning solutions. Physicians will be tasked regularly with clinical decision-making with the assistance of AI-driven predictions. Present-day physicians are not trained to incorporate the suggestions of such predictions on a regular basis nor are they knowledgeable in an ethical approach to incorporating AI in their practice and evolving standards of care. Medical schools do not currently incorporate AI in their curriculum due to several factors, including the lack of faculty expertise, the lack of evidence to support the growing desire by students to learn about AI, or the lack of Liaison Committee on Medical Education’s guidance on AI in medical education. Medical schools should incorporate AI in the curriculum as a longitudinal thread in current subjects. Current students should understand the breadth of AI tools, the framework of engineering and designing AI solutions to clinical issues, and the role of data in the development of AI innovations. Study cases in the curriculum should include an AI recommendation that may present critical decision-making challenges. Finally, the ethical implications of AI in medicine must be at the forefront of any comprehensive medical education.

## Introduction

Artificial intelligence (AI) and its applications hold great promise for solving many of health care’s global issues including making diagnoses, facilitating diagnostics, decision-making, big-data analytics, and administration [1,2]. AI has the potential to solve the global doctor shortage and bring access to health care to remote areas of the world [2].

Many fields of medicine have already seen benefit from the practical application of AI. Examples include the detection of atrial fibrillation, epilepsy seizures, and hypoglycemia, or the diagnosis of disease based on histopathological examination or medical imaging [3]. The use of AI is not limited to the fields of radiology or pathology; rather, those fields are indicators of the power of AI for image recognition, a singular form of data that transverses many fields spanning from primary care practice to urgent midsurgical decisions. Recent data show that every specialty in medicine is exploring the use of AI in assisting physicians [4]. Deep learning algorithms can make functional sense of increasing amounts of data used by individuals daily through wearables, smartphones, and other mobile monitoring sensors in different areas of medicine [3]. AI will continue to improve its capabilities to perform analysis and provide intelligent actionable recommendations on most forms of data [5]. It is expected that the future advancements in AI will permeate all aspects of medicine [6].

### Disruption

As AI continues to make advancements in health care, it is not without challenges. AI is met with resistance from physicians ill-equipped for such an evolution of clinical practice [3]. It is likely that physicians would benefit from the advancements of AI, but an understandable fear of replacement may prevent these opportunities. Furthermore, a lack of knowledge in AI can create skepticism in the trustworthiness of a machine learning prediction. This pushback may be preventing a large part of the health care sector from adapting to AI as other professional sectors continue to use AI solutions to advance their industries [3].

AI will change the dynamics of the traditional clinician-patient dyad to a much more ethically and emotionally complex clinician-AI-patient triad. This will dramatically alter the trust and accountability aspects with ethical, legal, and financial implications [7]. Physicians must be prepared for this great change [8].

Emerging technologies such as AI have the potential to disrupt labor markets maintained through traditional education programs. In order to be resilient to these market disruptions, physician training programs will require change [9]. The current undergraduate medical education (UME) curriculum is increasingly out of sync with the new needs of an evolving technology. Although most medical schools embrace change and strive to regularly update the components of the medical curriculum, a major overhaul is difficult to achieve and may be a hindrance to the implementation of AI in the curriculum. The path to significant curricular reform is difficult because of a variety of factors, including deeply embedded values and the accreditation process [10].

UME must begin to welcome the future and provide students access to a broader scope of health care through AI. Knowledge on data science, assessing algorithmic quality, and differentiating among different AI products are necessary components of medical education, which faculty must begin to incorporate. Medical schools must begin to teach and nurture unique human abilities that give physicians a competitive advantage over computers to establish an irreplaceable role in the future [9].

### What skills Do Physicians Need?

The practice of medicine is entering the age of AI in which the use of data to improve clinical decision-making will grow, bringing forth the need for skillful medicine-machine interaction [11]. Educating the next generation of physicians with the right techniques and adaptations to AI will enable them to become part of this emerging data science revolution [8]. Currently, there are different approaches for physicians to become accompanied to AI. There are physicians taking courses in data science, fellowship opportunities, and data scientists entering medical education programs. These represent a small fraction of the total physician population, and therefore a more integrative and forward approach in UME is necessary.

Medical professionals need to be sufficiently trained in AI, its advantages, and its potential to lower cost, improve quality, and expand access to health care. Of equal importance, physicians should be knowledgeable in its shortfalls such as transparency and liability. AI needs to be seamlessly integrated across the different aspects of the curriculum to achieve these goals [11].

When incorporating recommendations from AI solutions to a patient’s course of treatment, physicians should be capable to answer any concerns that patients may have. Perhaps even more importantly, physicians are responsible for ensuring that AI becomes a technology beneficial for patient care and not possibly a cause of harm. The technological revolution raises many challenges with regards to ethical considerations of AI-based implementation in health care. Minority exclusions in databases, issues with legal protections, and a decrease in humanistic touch, among other ethical issues, raise concern for an adaptation of AI in health care. These reasons bring forth the importance of acquiring sufficient knowledge and experience about AI, an obligation of high importance for future physicians [12].

Medical schools should take necessary steps to educate students with widespread knowledge of basic and clinical medicines along with data science, biostatistics, bioethical implications of AI, and evidence-based medicine. Part of a medical student’s training should include developing the abilities to distinguish correct information from rhetoric and to understand how to create and disseminate thoroughly validated, trustworthy information for patients and the public [12].

### Suggested Steps

Currently, the state of AI in medical education is in its infancy and speculative stages [13-16]. Previously, we have shown that the majority of published literature on the topic call for change in undergraduate medical education and that research is necessary to support curricular changes [17]. Even so, few have given thought to the steps that must be taken to create this change. This is expected because of the difficulty in implementing major curricular changes. Here, we provide an outline of the perceived difficulties and offer solutions to resolve these challenges (Table 1).

Medical school curricular changes are difficult to implement due to resistance to change. This resistance is justified through a lack of consensus on how to incorporate change and an already busy curriculum. For this reason, proposing additional courses or workload will likely be met with criticism from medical education faculty in the best interest of medical students. On a level of national infrastructure, these issues can be supported by leaders of medical education and organizations. These perceptions can be clarified easily through the addition of 1-3 questions on the annual Association of American Medical Colleges Graduation Questionnaire to gauge interest and ability over time from students. For example, the questionnaire can ask for agreement on whether AI should be taught during UME, what year of training it should be taught in, and how it should be incorporated. These can translate into accreditation requirements and drive change forward.

**Table 1:** Multitiered solutions to include AI^a^ in the medical education curriculum.

Levels and target areas of improvement		Examples
**National infrastructure**		
	AAMC^b^	Questionnaire, materials, and guidance
	LCME^c^	Minimal requirements and expert panels
	Multi-institutional research	Longitudinal research on attitudes and quality improvements
**Individual school**		
	Faculty expansion	Bioethics, bioinformatics, and medical AI experience
	Basic knowledge lessons	Introductory courses, benefits and pitfalls of AI, and ethics of AI
	Case-based learning	Multispecialty implications in previous cases and biostatistical implications
**Student specific**		
	Journal clubs or reading groups	Specialty-specific journals, health care systems journals, and AI in health care journals
	Use of AI in clinical setting	Tumor board, radiology rotation, and point of care ultrasound

^a^AI: artificial intelligence.

^b^AAMC: Association of American Medical Colleges.

^c^LCME: Liaison Committee on Medical Education.

A more overarching question is how undergraduate medical educators can unite to perform high-quality research on the incorporation of AI in the curriculum. Would individual school reports with differing standards in research and protocol do justice to a necessary change across all undergraduate medical education? A joint and united research effort from multiple medical schools would provide a multifaceted and diverse input on the issue and is necessary.

On an individual school level, investments of resources will be necessary to create improvements in the curriculum. A longitudinal AI thread throughout the UME should be advocated to solve time and content constraint issues. Courses teaching evidence-based medicine should incorporate an additional perspective of evaluating the input of AI. Medical school faculty may not be equipped to answer questions or discuss the role of AI in evidence-based medicine. Therefore, it is imperative to add clinician data scientists or computer science and engineering faculty from other schools to medical school teaching faculty.

Students learning public health sciences must be introduced to a background in AI in order to know what AI can and cannot do for their future research and practices. It is far too difficult to teach entire courses of AI in a medical school curriculum. An introductory lecture to AI in medicine is a necessity and should be advocated for. In most schools, the instructor will likely be a data scientist, but it is important that the instructor has teaching experience, is familiar with medical students, and is conversant with the role of AI in medicine. Preferably, the instructor will have already taught about the role of AI in medicine previously in a computer science course. Most importantly, instructors should have levels of competency in bioethics to address the expansive ethical issues AI has brought to health care.

Case-based learning and simulation learning can also incorporate AI-based recommendations in clinical scenarios. By integrating AI into cases and simulations, students can have exposure and familiarity with AI-based solutions. Collaborating and managing AI applications will require a deep understanding of probabilistic reasoning and ethical consideration from medical students [10]. These lessons should be taught from faculty with knowledge about the accuracy and interpretations of AI recommendations. It is likely that medical schools will need to hire additional faculty in this field to ensure quality delivery of AI in medicine content.

Another suggestion is for institutions to offer access to AI in web-based health care programs created at other institutions (eg, Stanford University’s Artificial Intelligence in Healthcare professional program). These programs are taught by faculty who are world-renowned experts at the interface of health care and AI, and the programs are available to medical students with a cost. Providing access to a program will enable medical schools to infuse new knowledge in a curriculum that could not be provided otherwise.

On an individual student level, students can help drive change in their education with a proactive engagement with AI. Previously, we have shown that most of the current literature agrees that medical students do not need to learn how to code and create AI tools, but they should understand how AI works and its limitations [17]. Students can read about AI advancements in health care in medical journals. AI research has a strong appearance in many leading journals, including medical AI-themed journals. Reading the current trends in AI in health care will inform and prepare students for the future of health care. When combined with evidence-based medicine learning at medical school, students will be able to assess the integrity of AI research.

Students can expose themselves to AI in the clinical setting as well. There are already reports of students receiving individual portable ultrasounds with AI-driven software to help advance their education [18,19]. Students can see AI applications in their radiology rotations and discuss its role in clinical decision-making with radiologists and clinicians across different specialties involved in patient care impacted by AI applications. AI integration will be specialty specific, but AI as a whole will likely be present across health care. For example, a tumor board, consisting of radiologists, pathologists, oncologists, and surgeons, is impacted by AI applications. Students can tailor their exposure toward fields of interests. The suggestions above apply to medical education as a whole and highlight the importance and room for improvement in general medical education. Specialty-specific AI topics would not be necessary for UME but perhaps in residency and beyond. Nevertheless, through a longitudinal curricular thread in AI, medical schools can expose their students to a wide variety of specialties using AI.

Lessons in decision-making with AI will apply to individuals across the spectrum of health care, both in city and rural settings. It is expected that AI will permeate various health care settings because of its potential to expand access to health care. Therefore, individual students should be prepared by learning about AI no matter what area of medicine they choose.

## A Methodological Approach

New medical curricular changes can have tremendous positive and negative impacts on medical students. Additionally, a change in curriculum such as the introduction of an entire novel topic is a difficult task. Therefore, it is important that medical education specialists across different regions work in unison to create and assess the implemented changes. Educational research is vital to assessing the effectiveness of different curricular reforms [20]. One suggestion to achieving such success is to begin a long-term study to measure the outcomes of different implementations of AI in the curriculum.

Research measuring student and faculty attitudes, skill level, and specific needs of AI in UME is crucial and urgent at this point in time. Further efforts to incorporate these suggestions should be measurable and have interpretable data to advance the implementation of AI in UME. A concerted multi-institutional study is a logical approach in order to achieve these goals.

Medical education deans need to gather to discuss and plan AI curricular reform. At the organizational level, medical education governing bodies must enact and promote these changes. The Liaison Committee on Medical Education should provide suggestions and guidelines of how to best incorporate AI to medical education through special committees.

In 2018, the American Medical Association called for “Research regarding the effectiveness of AI instruction in medical education on learning and clinical outcomes” [21]. Three years later, the available literature suggests UME has been slow to address this call [17]. Thus, further efforts should be made to advance this original call into practice.

## Conclusion

The current and future advancements of AI in medicine oblige undergraduate medical educators to act and implement AI in the curriculum. Longitudinal research plans are necessary to effectively study how to best achieve these curricular changes. Medical education governing bodies, medical education deans, and medical education researchers should begin to implement AI in the undergraduate medical education curriculum. Moving forward with collective agreement from these entities will ensure current students—our future physicians—receive adequate AI exposure.

## Abbreviations

AI: artificial intelligence
UME: undergraduate medical education
